# *Casearia tomentosa* fruit extracts exposed larvicidal activity and morphological alterations in *Culex quinquefasciatus* and *Aedes albopictus* under in vitro and semi field conditions

**DOI:** 10.1186/s13104-023-06663-x

**Published:** 2024-01-02

**Authors:** Priyanka Mandal, Goutam Chandra

**Affiliations:** Mosquito, Microbiology and Nanotechnology Research Units, Parasitology Laboratory, Department of Zoology, The University of Burdwan, Burdwan, West Bengal 713104 India

**Keywords:** *Casearia tomentosa*, *Culex quinquefasciatus*, *Aedes albopictus*, Phytochemicals, FTIR, GCMS, FE-SEM

## Abstract

Mosquitoes are notorious insects that transmit a wide range of infectious diseases, including zika, malaria, chikungunya, filariasis, and dengue. The overuse and incorrect application of synthetic pesticides to control mosquitoes has resulted in resistance development and environmental contamination, both of which have had a negative impact on human health. To address this issue, the larvicidal and pupicidal potential of acetone extract from *Casearia tomentosa* fruits was investigated. The extract was evaluated in a lab setting against all larval instars and pupa of *Culex quinquefasciatus* and *Aedes albopictus*, as well as against third instar larvae in a semi-field condition. Purified compounds through TLC were also tested against 3rd instar larvae of both mosquito and non-target organisms. The FT-IR and GC-MS analyses were used to characterise the extract. Morphological aberration caused by the acetone extract was observed using FESEM. The anal gills and respiratory siphon of both mosquitoes showed significant deformation from their normal state. 100 ppm was found to cause 100% larval mortality at 24 h of exposure in case of *Cx. quinquefasciatus* and at 72 h of exposure in *Ae. albopictus* larvae. After 72 h of exposure under in vitro conditions, the extract demonstrated considerable larvicidal activity with LC_50_ values of 38.33 and 47.56 against 3rd instar larvae of *Culex quinquefasciatus* and *Aedes albopictus*, respectively. The acetone extract can be considered as a highly effective mosquito larvicidal agent that is safe for the environment.

**Graphical abstract:**

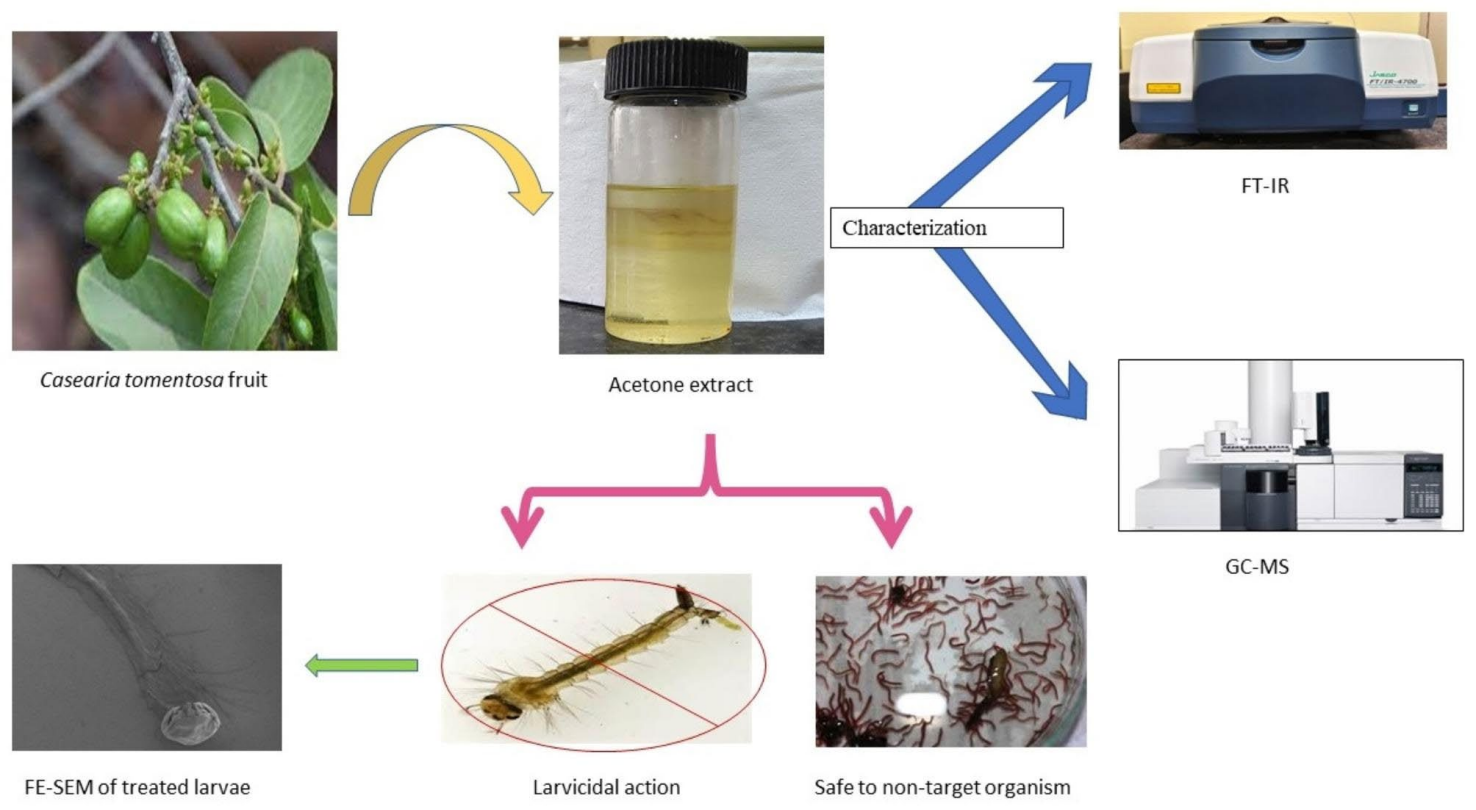

**Graphical representation depicting the larvicidal activity of the acetone extract of*Casearia tomentosa*fruits along with its effect on non-target organism**.

## Introduction

Mosquitoes are members of the Culicidae family and are the main insects responsible for the spread of many infectious diseases, including the protozoan disease malaria and helminthic diseases filariasis as well as the viral diseases dengue, chikungunya, yellow fever, rift valley fever, West Nile fever, zika [1, 2]. More than 100 species of the 3492 mosquito species known to exist in the world are capable of spreading a wide range of diseases to people and other vertebrates [3]. Millions of people die each year as a result of a wide variety of mosquito-borne diseases worldwide [4]. *Aedes albopictus* is a dengue vector, and it is estimated that about 50–100 million infections have occurred globally during the past 30 years [5]. Lymphatic filariasis is transmitted by *Culex quinquefasciatus* Say. One billion people from 72 different nations are at risk of filarial infection [6], and 40 million people are infected globally [5].

To promote the welfare of the society and the environment, it is crucial to prevent mosquito-borne diseases through controlling mosquito larvae present in aquatic habitats and adults present in air. Both are accomplished using synthetic chemical insecticides and repellents. Mosquitocides promise to effectively reduce dangerous mosquito populations, but sadly, the risks involved in using them have outweighed any positive outcomes. Non-selective pesticides also kill unintended plants and animals in addition to the intended targets. In addition, certain insects also develop genetic resistance to insecticides over time. However, the excess and improper uses of pesticides have led to the development of resistance and environmental contamination. Therefore, alternative environment conscious solutions must be developed immediately. As an alternative to synthetic mosquitocides, attempts are now being made to control the mosquito population using plant-derived compounds and phytochemicals that have effective mosquitocidal characteristics. Secondary metabolites in these natural products have larvicidal [7], pupicidal [8], adulticidal [9], repellent [10] and oviposition deterrence [11] effects on mosquitoes. These plant-based remedies are biodegradable, environment friendly, less damaging to non-target species, and relatively inexpensive [12].

*Casearia* species belonging in the Salicaceae family, has more than 160 species that have been identified and are of potential medicinal uses [13]. In South American and Asian countries, several *Casearia* species have been utilized as folk medicines for a very long period. Pharmacological investigations demonstrated that this genus had anti-inflammatory, anti-oxidant, antimicrobial, antibacterial, cytotoxic, antiulcer, hypoglycaemic, anti-mycobacterial and anti-snake venom properties [14–20]. One of the significant species in this genus is *Casearia tomentosa*, generally known as chilla. It is a small to medium-sized tree, found in India, North Australia, Pakistan, and Malaysia [21]. Various components of *C. tomentosa* are said to have therapeutic uses for treating ulcers, edema, fissures, colic discomfort in the abdomen, malaria, tonsillitis pain, wounds, hypertension, piles, ringworm, diabetes, and in severe bone fractures as a plaster [22–25]. However, no information has been found about its mosquito larvicidal property untill now. So, it is a vital step to investigate its larvicidal property.

The purpose of this study was to assess the larvicidal and pupicidal effects of solvent extracts of *C. tomentosa* fruits in laboratory conditions as well as the larvicidal activities of acetone extract against *Culex quinquefasciatus* and *Aedes albopictus* in semi-field conditions and to evaluate adverse effects of the active compounds on both target and non-target organisms (*Chironomus circumdatus* and *Toxorhynchites splendens* larvae), and also to investigate any morphological aberration derived from the acetone extract on both the mosquito species under FESEM study.

## **Materials and methods**

### Collection and rearing of mosquito

Juveniles of *Cx. quinquefasciatus* were collected with the scooping and dipping method [26] from some stagnant drains of Burdwan (23°16’, 87°54’), West Bengal, India. Larvae of *Ae. albopictus* were collected from Burdwan University Campus from previously placed ovitraps. Immatures were then transferred and reared in separate trays in preferrable habitat conditions. Larval colonies were maintained at 26 ± 2 °C, 70 ± 5% relative humidity and 14:10 h of light and dark cycles. Larvae were fed with commercial dog biscuits and yeast at a ratio of 3:2 [27].

### Collected plant species

Fresh and mature *C. tomentosa* fruits were obtained randomly from plants near Pandaveshwar; Paschim Barddhaman in West Bengal, India (23°43′N, 87°16′E) during the months of June to August. The plant was identified by Dr. Ambarish Mukherjee and a voucher specimen GCRKM/2017/S010 was kept at the Mosquito, Microbiology and Nanotechnology Research Units, Parasitology Laboratory, Department of Zoology, The University of Burdwan.

### Solvent extract preparation

The fruits were rinsed with distilled water, and then dried on tissue papers. Fruits were cut into very small pieces (2 cm × 1.5 cm × 1.5 cm) and air dried under shade for about 20 days. The dried fruits were run into Soxhlet apparatus with two solvents one with mid-range of polarity that is acetone and another with non-polar range that is water. 150 gm of dried fruit pieces were loaded in the thimble of Soxhlet apparatus with 1500 ml of acetone in still pot at a ratio of 1:10 [1] for acetone extraction. Then same process was followed with distilled water solvent. Extractives were poured in the glass beaker and made concentrated through rotary evaporator. The concentrated semi solid extractives were preserved in refrigerator at 4 °C for bioassay tests.

### Larvicidal bioassay

The bioassay experiments were carried out in accordance with World Health Organization (WHO) guidelines [28]. Twenty-five larvae of each instar (1st, 2nd, 3rd, 4th ) of *Cx. quinquefasciatus* and *Ae. albopictus* were transferred separately in 250 ml glass beaker containing 100 ml of water. Graded concentrations of solvent extractive (25, 50, 75, 100, 125 ppm) were then applied to each beaker. Twenty-five larvae of each larval instar of each mosquito species were introduced to a beaker filled with 100 ml of tap water on each experiment day as a set of control. Each experiment was triplicated on three different days with three sets of control. Larval mortality was recorded at 24 h, 48 and 72 h after initiation of the experiment. Larvae were assumed to be dead when they failed to respond after inserting needle into the siphon or cervical region or when they were unable to reach the water surface [29].

#### Pupicidal bioassay

A graded concentration of acetone extract (50 ppm, 100 ppm, 150 ppm, 200 ppm, and 250 ppm) was made and put into individual beakers with 100 ml of distilled water. In each experimental beaker, 25 early pupae of each mosquito species that is *Cx. quinquefasciatus* and *Ae. albopictus* were placed. The death rate of pupa was noted at 24-, 48-, and 72-hours following exposure. Three control sets were used in the three replications of the study.

#### Semi field experiment

Semi field experiment was done according to the guidelines of WHO [30] and Karunamoorthi et al. [31] against both the mosquito, *Cx. quinquefasciatus* and *Ae. albopictus* separately. Five glass jars for each mosquito species were placed in the garden of the department and each container was filled with 5000 ml of their preferable habitat water. Then graded concentration of 50 ppm to 250 ppm of acetone extract was applied to each container. These sets were repeated three times. 5000 ml of habitat water were placed in each of three glass containers for each mosquito species as a control. In each glass container, 100 third-instar larvae were released and any larval mortality was noted after 24 h, 48 h, and 72 h. To prevent other mosquitoes from laying their eggs in these glass containers, a mosquito net was placed over every container.

**Phytochemical analysis test** Qualitative phytochemical analysis of the plant extract was performed according to Harborne [32], Trease and Evans [33] and Sofowara [34] with minor modifications.The phytochemicals included under study were saponins, terpenoids, alkaloid, steroids, tannin, flavonoids and anthocyanin.

#### Test for tannin

5–10 drops of FeCl3 were combined with 2 ml of acetone extract in accordance with the Ferric chloride test. The presence of tannins is indicated by the emergence of bluish-black colour.

#### Test for steroid and terpenoid

1 ml of acetone extract was acidified with 1 N glacial acetic acid, and then 1 ml of concentrated 4 N H2SO4 was introduced via the test tube wall within the ice chamber. When colour turns brown, steroids are present; when colour turns green, terpenoids are present.

#### Test for flavonoid

A little amount of zinc was added after 1 ml of acetone extract and 10 drops of 0.5 N HCL were combined, in accordance with the Zinc Hydrochloride Test. The precipitation of a pink or reddish-pink colour indicates the presence of flavonoids.

#### Test for saponin

5 ml of crude extract were mixed with a few drops of NaHCO3, then thoroughly shaken. After that, the sample was left alone for 3 min. The presence of saponin is indicated by the production of stable foam that resembles honeycomb.

#### Test for coumarin

3 ml of 10% NaOH was added to 2 ml of acetone extract. The rapid change in colour of acetone extract to yellow indicates the presence of coumarin in the extract.

#### Test for alkaloid

Using Mayer’s assays, the existence of alkaloids was investigated. To make Mayer’s reagent, mix 1.36 g of HgCl2 and 5 g of KI in 100 ml of distilled water. The acetone extract was first made acidic by adding glacial acetic acid. Mayer’s reagent was applied to 1 ml of the acidified crude extract. This test indicates the presence of alkaloids when a pale-yellow precipitate appears.

#### Test for anthocyanin

To 2 ml of acetone extract, 1 ml of 2 N HCL and 1 ml of NH3 were added. The extract contains anthocyanins if the pink-red colour immediately changes to a blue-violet hue.

### Isolation of the active compound through thin-layer chromatography

The acetone extract was chromatogrammed in a previously silica (Silica -Gel “G”, Merck, India) layered TLC plate (20 cm × 20 cm) in a chamber saturated with solvents (methanol, acetone and ethyl acetate) in increasing polarity. After running the solvent, the plates were picked up from the chamber, air dried at room temperature and analysed under UV light chamber to find the bioactive bands accurately. From this solvent ratio two different bands were attained. Each spot was then scrapped and collected separately according to their Rf values. The collected bands were then mixed and dissolved in acetone, and filtered by Whatman No 1 filter paper to get active component, discarding the silica gel fraction. In order to identify whether the bio active component present in the scrapings showed larval toxicity, acetone was evaporated at room temperature and the dried compound was subjected to the bioassay experiment with the third-instar larvae of *Cx. quinquefasciatus* and *Ae. albopictus*. The dried compound was further used for FT-IR and GC-MS analyses.

### FT-IR and GC-MS analyses of the active compound

In order to identify the functional groups, different bonds, chemical composition of the compound present in the plant extract, FT-IR spectroscopy was used. FT-IR spectroscopy was done with the positive response band compound in FT-IR spectrophotometer (JASCO FT-IR Model-4700) at room temperature (25 ± 5 °C) with a scanning range from 400 to 4500 cm^− 1^ to identify the functional groups of acetone extract. The peaks have been analysed using KnowItAll software (serial no. 107733-00001F44).

GC-MS analyses of the sample was done and utilised for chemical characterization of the bioactive component using GC-MS apparatus (Model: Clarus 680 GC). The Software used in the system is TurboMass Version 6.4.2. The capillary column used is ‘Elite- 5MS’ having dimensions length 60 m, ID 0.25 mm and film thickness 0.25 µm and the stationary phase is 5% diphenyl 95% dimethyl polysiloxane. At a flow rate of 1 ml/min, helium gas (99.99%) was utilised as the carrier gas (i.e., mobile phase). The run time was about 39 minutes. An 8-minute solvent delay was maintained. NIST-2014, a data analysis library, was used to assess the peaks.

### Larvicidal bioassay with active compound

Larval mortality was also tested with two bio active compounds of acetone extract isolated through thin-layer chromatography (TLC) according to similar methodologies of WHO, 2005. Graded concentrations of bioactive compound (5ppm, 10 ppm, 15 ppm and 20 ppm) were given to each beaker containing twenty-five 3rd instar larvae of *Cx. quinquefasciatus* and *Ae. albopictus* separately. Larval mortality was noted after 24 h, 48 and 72 h respectively. Each set was triplicated on three different days with a set of control.

### Test on non-target organisms

Effect of acetone extract derived active compounds were also tested against *Chironomus circumdatus* larvae (chironomid) and *Toxorhynchites splendens* larvae which share same habitat with larvae of *Cx. quinquefasciatus* and *Ae. albopictus* respectively with LC_50_ values (at 24 h) of third instar of both the mosquito species according to the procedure of Suwannee et al. [35]. Mortality and other behavioural abnormalities (reduced swimming activity and sluggishness) were observed after 24 h, 48 and 72 h of exposure.

### Statistical analysis

The average larval mortality data were calculated using Abbot’s algorithm [36]. Regression equations (Y = mortality; X = concentrations) and regression coefficient values, LC_50_ and LC_90_ values as well as other statistics at a 95% confidence limit with an upper and lower confidence limit was calculated by using the software STATPLUS PRO 2009, MS EXCEL 2009. The normalization of data (that is Shapiro-Wilk) and three-way analysis of variance (ANOVA) test was performed with the result of solvent extract with the help of SPSS16.0 (Statistical Package for Social Sciences) software version 16.0. Statistical significance was taken into account when p-value ≤ 0.05.

### SEM study of the larvae

The treated dead larvae and alive control larvae of both mosquito species were collected from different beakers. The samples were then dehydrated by a series of alcohol (50%, 70%, 90% and 100%) for 15–20 min each. The samples were dried in Critical Point Dryer (CPD) for about 8–10 min. Before coating by gold using a sputter coater the larvae were mounted on an aluminium stub with carbon tape. The drying was achieved with utilizing a vacuum of 1,250 psi and a heating temperature of 37 °C. The coating was performed for 180 s. Morphology of both control and treated larvae were observed by highly advanced Field emission scanning electron microscope (Gemini, ZEISS, Sigma 300).

## Results

When the two extracts were compared, the acetone extract exhibited much more mortality than the water extract. *Cx. quinquefasciatus* and *Ae. albopictus* exhibited 100% larval morality in the presence of acetone extract after 72 h of exposure at concentrations of 100 ppm and 125 ppm, respectively. However, after being exposed to 125 ppm concentrations for 72 h, *Cx. quinquefasciatus* and *Ae. albopictus* only displayed 70.67% and 65.33% in the case of the water extract. Results of larvicidal bioassay of water and acetone extracts of *C. tomentosa* fruit are tabulated in Table 1.

**Table 1:** Dose response larvicidal bioassay using two solvent (acetone and water) extracts of *Casearia tomentosa* fruits against 3rd instar larvae of *Culex quinquefasciatus* and *Aedes albopictus*

Mosquito species	Solvent extract used	Concentration (ppm)	Percent Mortality (Mean ± SE)		
			24 h	48 h	72 h
*Culex quinquefasciatus*	Acetone	25	28.00 ± 0.58	46.67 ± 0.88	62.67 ± 0.67
		50	54.67 ± 0.33	66.67 ± 0.88	76.00 ± 0.58
		75	70.67 ± 0.33	81.33 ± 0.67	93.33 ± 0.33
		100	82.67 ± 0.88	94.67 ± 0.33	100.00 ± 0.00
		125	97.33 ± 0.33	100.00 ± 0.00	100.00 ± 0.00
		Control	00.00 ± 0.00	00.00 ± 0.00	00.00 ± 0.00
	Water	25	10.67 ± 0.33	21.33 ± 0.33	34.67 ± 0.33
		50	18.67 ± 0.33	30.67 ± 0.33	49.33 ± 0.33
		75	29.33 ± 0.33	40.00 ± 0.58	57.33 ± 0.33
		100	40.00 ± 0.58	49.33 ± 0.33	61.33 ± 0.67
		125	54.67 ± 0.33	62.67 ± 0.33	70.67 ± 0.33
		Control	00.00 ± 0.00	00.00 ± 0.00	00.00 ± 0.00
*Aedes albopictus*	Acetone	25	24.00 ± 0.58	33.33 ± 0.67	45.33 ± 0.88
		50	41.33 ± 0.67	52.00 ± 0.58	62.67 ± 0.33
		75	53.33 ± 0.33	69.33 ± 0.67	78.67 ± 0.33
		100	73.33 ± 0.88	85.33 ± 0.88	89.33 ± 0.67
		125	90.67 ± 0.67	97.33 ± 0.33	100.00 ± 0.00
		Control	00.00 ± 0.00	00.00 ± 0.00	00.00 ± 0.00
	Water	25	8.00 ± 0.00	17.33 ± 0.33	30.67 ± 0.33
		50	14.67 ± 0.33	26.67 ± 0.33	45.33 ± 0.88
		75	25.33 ± 0.33	34.67 ± 0.33	50.67 ± 0.67
		100	36.00 ± 0.58	46.67 ± 0.67	56.00 ± 0.58
		125	48.00 ± 0.58	58.67 ± 0.33	65.33 ± 0.33
		Control	00.00 ± 0.00	00.00 ± 0.00	00.00 ± 0.00

The first instar of *Cx. quinquefasciatus* larvae showed 100% mortality at a concentration of 100 ppm of acetone extract after 24 h of exposure; the second and third instars also showed 100% mortality at the same concentration after 72 h of exposure; and the fourth instars demonstrated 100% mortality at a concentration of 125 ppm after 48 h of exposure. After 72 h of exposure, acetone extract in *Ae. albopictus* revealed 100% mortality at 100 ppm in the first and second instar and at 125 ppm in the third and fourth instar. With longer exposure times, the mortality rate for each larval instar climbed. Percent mortalities of acetone extractive against *Cx. quinquefasciatus* and *Ae. albopictu*s are presented in Tables 2 and 3 respectively. Log probit and regression analysis of acetone extract against *Cx. quinquefasciatus* and *Ae. albopictu*s is presented in Table 4. Log-probit analysis revealed a reversely proportional association (95% confidence level) between the LC values and the exposure period. The lowest recorded values of LC50 and LC90 in *Cx. quinquefasciatus* were 35.05 and 53.91, respectively. The first instar larvae of *Ae. albopictus* had the lowest LC50 and LC90, which were obtained at 37.69 and 67.33. *C. tomentosa* had the most potent larvicidal activity against Ae. albopictus and *Cx. quinquefasciatus* larvae in their first instar. The mortality rate (Y) and the extract concentration (x) had a positive relationship, with a regression coefficient (R2) in both regression analyses. The fruits of *C. tomentosa* acetone extractive demonstrated remarkable pupicidal activity. 100% pupal mortality was recorded at 200 ppm concentration after 72 h of exposure in case of *Cx quinquefasciatus* and at 250 ppm concentration at 48 h of exposure in *Ae albopictus* (Table 5).

**Table 2:** Dose response instar specific larvicidal bioassay using acetone extract of *Casearia tomentosa* on *Culex quinquefasciatus*

Larval instars	Concentration (ppm)	Percent Mortality (Mean ± SE)		
		24 h	48 h	72 h
First	25	34.67 ± 0.88	53.33 ± 0.33	70.67 ± 0.33
	50	54.67 ± 0.33	77.33 ± 0.33	85.33 ± 0.33
	75	80.00 ± 0.58	93.33 ± 0.67	98.67 ± 0.33
	100	100.00 ± 0.00	100.00 ± 0.00	100.00 ± 0.00
	125	100.00 ± 0.00	100.00 ± 0.00	100.00 ± 0.00
	Control	00.00 ± 0.00	00.00 ± 0.00	00.00 ± 0.00
Second	25	33.33 ± 0.33	52.00 ± 0.58	70.67 ± 0.67
	50	50.67 ± 0.33	65.33 ± 0.33	82.67 ± 0.33
	75	66.67 ± 0.88	86.67 ± 0.33	97.33 ± 0.33
	100	88.00 ± 0.58	98.67 ± 0.33	100.00 ± 0.00
	125	100.00 ± 0.00	100.00 ± 0.00	100.00 ± 0.00
	Control	00.00 ± 0.00	00.00 ± 0.00	00.00 ± 0.00
Third	25	28.00 ± 0.58	46.67 ± 0.88	62.67 ± 0.67
	50	54.67 ± 0.33	66.67 ± 0.88	76.00 ± 0.58
	75	70.67 ± 0.33	81.33 ± 0.67	93.33 ± 0.33
	100	82.67 ± 0.88	94.67 ± 0.33	100.00 ± 0.00
	125	97.33 ± 0.33	100.00 ± 0.00	100.00 ± 0.00
	Control	00.00 ± 0.00	00.00 ± 0.00	00.00 ± 0.00
Fourth	25	18.67 ± 0.33	36.00 ± 1.00	46.67 ± 0.88
	50	49.33 ± 0.33	60.00 ± 0.58	72.00 ± 0.58
	75	66.67 ± 0.33	76.00 ± 0.58	85.33 ± 0.33
	100	86.67 ± 0.88	90.67 ± 0.67	98.67 ± 0.33
	125	88.00 ± 0.58	100.00 ± 0.00	100.00 ± 0.00
	Control	00.00 ± 0.00	00.00 ± 0.00	00.00 ± 0.00

**Table 3:** Dose response instar specific larvicidal bioassay using acetone extract of *Casearia tomentosa* on *Aedes albopictus*

Larval instars	Concentration (ppm)	Percent Mortality (Mean ± SE)		
		24 h	48 h	72 h
First	25	29.33 ± 0.33	40.00 ± 0.58	53.33 ± 0.33
	50	48.00 ± 0.58	58.67 ± 0.33	76.00 ± 0.58
	75	65.33 ± 0.88	77.33 ± 0.33	89.33 ± 0.33
	100	81.33 ± 0.67	89.33 ± 0.67	100.00 ± 0.00
	125	100.00 ± 0.00	100.00 ± 0.00	100.00 ± 0.00
	Control	00.00 ± 0.00	00.00 ± 0.00	00.00 ± 0.00
Second	25	26.67 ± 0.33	34.67 ± 0.33	50.67 ± 0.67
	50	45.33 ± 0.88	57.33 ± 0.88	73.33 ± 0.33
	75	62.67 ± 0.67	74.67 ± 0.67	86.67 ± 0.33
	100	78.67 ± 0.67	90.67 ± 0.67	100.00 ± 0.00
	125	97.33 ± 0.33	100.00 ± 0.00	100.00 ± 0.00
	Control	00.00 ± 0.00	00.00 ± 0.00	00.00 ± 0.00
Third	25	24.00 ± 0.58	33.33 ± 0.67	45.33 ± 0.88
	50	41.33 ± 0.67	52.00 ± 0.58	62.67 ± 0.33
	75	53.33 ± 0.33	69.33 ± 0.67	78.67 ± 0.33
	100	73.33 ± 0.88	85.33 ± 0.88	89.33 ± 0.67
	125	90.67 ± 0.67	97.33 ± 0.33	100.00 ± 0.00
	Control	00.00 ± 0.00	00.00 ± 0.00	00.00 ± 0.00
Fourth	25	21.33 ± 0.33	29.33 ± 0.33	38.67 ± 0.67
	50	38.67 ± 0.67	45.33 ± 1.20	56.00 ± 0.58
	75	54.67 ± 0.67	62.67 ± 0.67	69.33 ± 0.33
	100	69.33 ± 0.33	77.33 ± 0.33	82.67 ± 0.88
	125	77.33 ± 0.33	86.67 ± 0.33	100.00 ± 0.00
	Control	00.00 ± 0.00	00.00 ± 0.00	00.00 ± 0.00

**Table 4:** Log probit and regression analyses of larval mortality treated with acetone extract of *Casearia tomentosa* fruit against *Culex quinquefasciatus* and *Aedes albopictus*

Larva	Larval Instars	Period of Exposure	LC_50_	LC_90_	Regression	R^2^- value
*Culex quinquefasciatus*	First	24	49.52	78.72	y = 0.704x + 21.067	R² = 0.9362
		48	36.10	60.45	y = 0.464x + 50.000	R² = 0.8512
		72	36.54	53.91	y = 0.2933x + 68.933	R² = 0.8071
	Second	24	53.04	100.25	y = 0.6827x + 16.533	R² = 0.9949
		48	43.83	73.55	y = 0.5173x + 41.733	R² = 0.9342
		72	35.05	56.99	y = 0.304x + 67.333	R² = 0.8479
	Third	24	48.75	110.86	y = 0.6667x + 16.667	R² = 0.9741
		48	41.72	82.21	y = 0.5387x + 37.467	R² = 0.9631
		72	38.33	64.04	y = 0.3947x + 56.80	R² = 0.8936
	Fourth	24	51.59	126.57	y = 0.704x + 9.0667	R² = 0.9262
		48	46.23	91.41	y = 0.6347x + 24.933	R² = 0.9731
		72	39.67	73.12	y = 0.5333x + 40.533	R² = 0.9114
*Aedes albopictus*	First	24	55.29	109.23	y = 0.6987x + 12.40	R² = 0.9994
		48	46.35	93.27	y = 0.6027x + 27.867	R² = 0.9831
		72	37.69	67.33	y = 0.4693x + 48.533	R² = 0.8913
	Second	24	57.21	117.91	y = 0.6987x + 9.7333	R² = 0.9994
		48	47.77	93.96	y = 0.656x + 22.267	R² = 0.9791
		72	39.64	70.03	y = 0.5013x + 44.533	R² = 0.9098
	Third	24	61.86	145.69	y = 0.6613x + 6.9333	R² = 0.9951
		48	50.10	108.97	y = 0.6453x + 19.067	R² = 0.9938
		72	47.56	93.33	y = 0.544x + 34.40	R² = 0.986
	Fourth	24	65.06	203.99	y = 0.656x + 22.267	R² = 0.9791
		48	56.31	147.03	y = 0.5867x + 16.267	R² = 0.9895
		72	49.63	105.93	y = 0.5973x + 24.533	R² = 0.9971

**Table 5:** Dose response pupicidal bioassay using acetone extract of *Casearia tomentosa* fruits on *Culex quinquefasciatus* and *Aedes albopictus*

Mosquito species	Concentration (ppm)	Percent Mortality (Mean ± SE)		
		24 h	48 h	72 h
*Culex quinquefasciatus*	50	52.67 ± 0.88	58.33 ± 1.42	80.00 ± 0.57
	100	64.67 ± 0.88	70.67 ± 0.88	87.33 ± 0.88
	150	73.00 ± 1.15	81.00 ± 0.57	96.33 ± 0.33
	200	84.00 ± 0.57	94.00 ± 0.57	100.00 ± 0.00
	250	92.33 ± 0.33	100.00 ± 0.00	100.00 ± 0.00
	Control	0.00 ± 0.00	0.00 ± 0.00	0.00 ± 0.00
*Aedes albopictus*	50	47.33 ± 1.20	57.00 ± 0.57	78.67 ± 0.88
	100	60.00 ± 1.00	69.33 ± 0.88	84.33 ± 0.66
	150	71.67 ± 1.20	80.00 ± 0.57	93.00 ± 0.57
	200	82.67 ± 1.45	93.33 ± 1.20	99.67 ± 0.33
	250	91.00 ± 1.15	100.00 ± 0.00	100.00 ± 0.00
	Control	0.00 ± 0.00	0.00 ± 0.00	0.00 ± 0.00

The Shapiro-Wilk test was used to determine whether the acetone extract mortality was normal in respect with different time, concentration, and instar. The data is considered normal if the sig. value is higher than 0.05. (Table 6).

**Table 6:** Shapiro Wilk test on larval mortality of *Culex quinquefasciatus* and *Aedes albopictus* treated with acetone extractive of *Casearia tomentosa* fruits

Mortality	Mosquito species	variables		Shapiro-Wilk		
				Statistic	df	Sig.
	*Culex quinquefasciatus*	Hour	24	0.931	20	0.159
			48	0.885	20	0.021
			72	0.817	20	0.002
		Instars	1st	0.806	15	0.004
			2nd	0.868	15	0.031
			3rd	0.908	15	0.127
			4th	0.923	15	0.214
		Concentration (ppm)	25	0.957	12	0.739
			50	0.939	12	0.481
			75	0.933	12	0.413
			100	0.793	12	0.008
			125	0.417	12	0.000
	*Aedes albopictus*	Hour	24	0.960	20	0.535
			48	0.937	20	0.206
			72	0.899	20	0.039
		Instars	1st	0.914	15	0.155
			2nd	0.921	15	0.200
			3rd	0.955	15	0.610
			4th	0.977	15	0.945
		Concentration (ppm)	25	0.953	12	0.688
			50	0.943	12	0.533
			75	0.963	12	0.829
			100	0.962	12	0.809
			125	0.670	12	0.000

According to a three-way ANOVA analysis, larval mortality of both *Cx. quinquefasciatus* and *Ae. albopictu*s was significantly influenced by larval instars, the concentration of the extract and time of exposure. During complex interactions with three variables, the interaction between instar and hour, instar and concentrations, hour and concentration and all the three factors together were found to significantly affect the larval mortalities (Tables 7 and 8).

**Table 7:** Three-way ANOVA analysis on larval mortality of *Culex quinquefasciatus* treated with acetone extractive of *Casearia tomentosa* fruits

Source	Type III sum of square	df	Mean square	F	Significance
Corrected model	5547.311^a^	59	94.022	132.219	0.000
Intercept	68133.356	1	68133.356	95812.531	0.000
Instar	198.289	3	66.096	92.948	0.000
Hour	717.478	2	358.739	504.477	0.000
concentration	4277.644	4	1069.411	1503.859	0.000
Instar * hour	7.544	6	1.257	1.768	0.111
Instar *concentration	64.044	12	5.337	7.505	0.000
Hour * concentration	227.522	8	28.440	39.994	0.000
Instar * hour * concentration	54.789	24	2.283	3.210	0.000
Error	85.333	120	0.711		
Total	73766.000	180			
Corrected total	5632.644	179			

a. R Squared = 0.970 (Adjusted R Squared = 0.955)

**Table 8:** Three-way ANOVA analysis on larval mortality of *Aedes albopictus* treated with acetone extractive of *Casearia tomentosa* fruits

Source	Type III sum of square	df	Mean square	F	Significance
Corrected model	6198.000^a^	59	105.051	64.980	0.000
Intercept	52020.000	1	52020.000	32177.320	0.000
Instar	335.111	3	111.704	69.095	0.000
Hour	612.033	2	306.017	189.289	0.000
concentration	5083.611	4	1270.903	786.125	0.000
Instar * hour	10.722	6	1.787	1.105	0.363
Instar *concentration	21.056	12	1.755	1.085	0.379
Hour * concentration	68.689	8	8.586	5.311	0.000
Instar * hour * concentration	66.778	24	2.782	1.721	0.030
Error	194.000	120	1.617		
Total	58412.000	180			
Corrected total	6392.000	179			

a.R Squared = 0.970 (Adjusted R Squared = 0.955)

The acetone extractive of *C.tomentosa* fruits showed promising larvicidal properties against third instar larvae of both *Cx, quinquefasciatus* and *Ae. albopictus*, as shown in Table 9 under simulated semi-field conditions. 100% larval death was observed in *Cx. quinquefasciatus* after 48 h of exposure and in *Ae. albopictus* after 72 h of exposure at 200 and 250 ppm concentrations of the aforementioned extractive, respectively.

**Table 9:** Larvicidal activity of acetone extractive of *Casearia tomentosa* against *Culex quinquefasciatus* and *Aedes albopictus* larvae under simulated semi-field condition

Larva	Time of exposure (hour)	Concentration (ppm)	Percent mortality (Mean ± S.E)		
			Day 1	Day 2	Day 3
*Culex quinquefasciatus*	24 h	50	34.67 ± 0.58	33.67 ± 1.15	30.67 ± 0.88
		100	52.33 ± 0.67	55.33 ± 1.58	53.33 ± 0.58
		150	69.33 ± 0.88	66.50 ± 0.67	66.50 ± 0.67
		200	83.33 ± 0.67	82.67 ± 0.33	79.50 ± 0.33
		250	92.67 ± 1.58	95.33 ± 0.88	89.33 ± 1.58
		Control	5.33 ± 0.33	6.50 ± 0.58	5.67 ± 0.33
	48 h	50	56.67 ± 1.15	52.67 ± 1.58	53.67 ± 0.33
		100	68.50 ± 0.67	65.33 ± 1.15	62.50 ± 0.33
		150	83.00 ± 1.00	80.67 ± 0.88	77.33 ± 0.00
		200	95.33 ± 0.88	95.50 ± 1.15	89.67 ± 1.58
		250	100.00 ± 0.00	100.00 ± 0.00	98.50 ± 0.56
		Control	7.67 ± 0.88	8.33 ± 0.33	8.67 ± 0.33
	72 h	50	65.33 ± 1.15	67.00 ± 0.00	65.33 ± 0.33
		100	79.33 ± 0.88	81.50 ± 0.67	80.33 ± 0.88
		150	90.67 ± 1.58	92.67 ± 0.88	90.50 ± 1.15
		200	100.00 ± 0.00	100.00 ± 0.00	98.67 ± 0.33
		250	100.00 ± 0.00	100.00 ± 0.00	100.00 ± 0.00
		Control	10.33 ± 0.67	12.67 ± 0.88	11.00 ± 1.00
*Aedes albopictus*	24 h	50	23.67 ± 0.88	25.33 ± 0.67	26.33 ± 0.67
		100	39.33 ± 0.67	41.67 ± 0.33	39.67 ± 0.88
		150	52.33 ± 0.33	53.67 ± 0.88	53.50 ± 0.33
		200	69.33 ± 1.58	69.33 ± 1.15	69.67 ± 1.15
		250	82.67 ± 1.15	80.33 ± 0.88	80.33 ± 1.58
		Control	4.67 ± 0.33	5.33 ± 0.33	5.67 ± 0.67
	48 h	50	43.33 ± 1.58	40.67 ± 1.58	42.33 ± 0.33
		100	58.33 ± 1.15	56.33 ± 1.15	56.67 ± 0.88
		150	70.00 ± 1.00	69.33 ± 0.88	68.50 ± 0.67
		200	82.33 ± 0.33	83.67 ± 0.67	85.33 ± 1.58
		250	96.00 ± 1.00	96.33 ± 0.33	97.67 ± 1.15
		Control	8.67 ± 0.33	8.00 ± 1.00	9.33 ± 0.33
	72 h	50	57.67 ± 1.15	55.67 ± 0.88	56.50 ± 0.67
		100	69.33 ± 0.88	70.50 ± 0.67	70.33 ± 0.88
		150	81.67 ± 1.58	80.67 ± 1.58	81.50 ± 0.33
		200	94.67 ± 0.67	95.33 ± 1.15	95.33 ± 0.88
		250	100.00 ± 0.00	100.00 ± 0.00	100.00 ± 0.00
		Control	11.67 ± 0.67	9.67 ± 0.33	9.50 ± 0.33

Percent mortality of both the mosquito species treated with TLC fraction of acetone extract is depicted in Table 10. 100% larval death was observed in case of 20 ppm concentration of active compound at 48 and 72 h of exposure in *Cx, quinquefasciatus* and *Ae. albopictus* respectively. No significant larval mortality or abnormality was observed with acetone extractive derived active compounds of *C. tomentosa* fruits against both *Chironomus circumdatus* and *Toxorhynchites splendens* (Table 11).

**Table 10:** Percent mortality of third instar larvae of *Culex quinquefasciatus* and *Aedes albopictus* treated with TLC fraction of acetone extract of *Casearia tomentosa* fruits under laboratory condition

Mosquito species	Concentration (ppm)	Percent Mortality (Mean ± SE)		
		24 h	48 h	72 h
*Culex quinquefasciatus*	5	45.33 ± 0.88	58.67 ± 0.33	69.33 ± 0.33
	10	58.67 ± 0.67	72.00 ± 0.58	78.67 ± 0.67
	15	74.67 ± 0.33	85.33 ± 0.33	89.33 ± 0.33
	20	85.33 ± 0.33	100.00 ± 0.00	100.00 ± 0.00
	Control	00.00 ± 0.00	00.00 ± 0.00	00.00 ± 0.00
*Aedes albopictus*	5	30.67 ± 0.67	41.33 ± 0.33	62.67 ± 0.33
	10	48.00 ± 0.58	58.67 ± 0.67	74.67 ± 0.33
	15	62.67 ± 0.33	80.00 ± 0.58	88.00 ± 0.58
	20	77.33 ± 0.33	90.67 ± 0.67	100.00 ± 0.00
	Control	00.00 ± 0.00	00.00 ± 0.00	00.00 ± 0.00

**Table 11:** Effect of acetone extract derived active compound of *Casearia tomentosa* fruit on a few non-target organisms at laboratory condition

Name of the organism	Percent Mortality (Mean ± SE)		
	24 h	48 h	72 h
Control	0.00 ± 0.00	0.00 ± 0.00	0.00 ± 0.00
*Chironomus circumdatus*	0.00 ± 0.00	0.00 ± 0.00	2.33 ± 0.33
Control	0.00 ± 0.00	0.00 ± 0.00	0.00 ± 0.00
*Toxorhynchites splendens*	0.00 ± 0.00	1.33 ± 0.33	7.33 ± 0.58

**Fig. 1:**
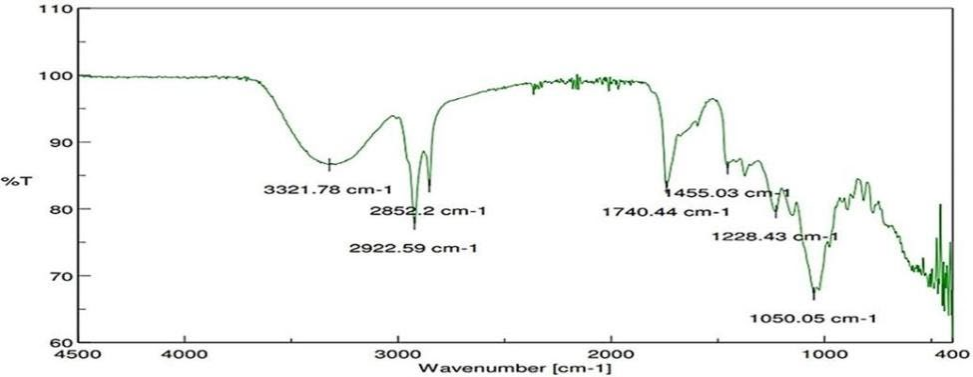
Interpretation of FT-IR spectra of acetone extract of *Casearia tomentosa* fruit

**Fig. 2:**
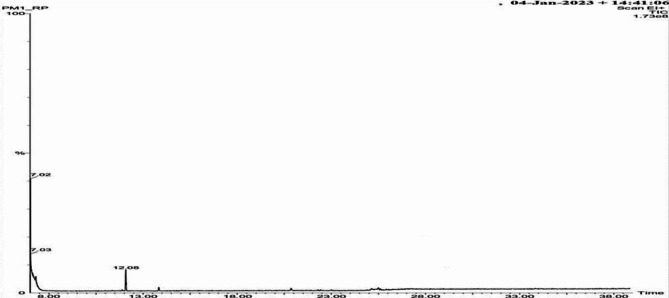
GC-MS chromatogram of TLC fraction obtained from acetone extract of *Casearia tomentosa* fruit

**Fig. 3:**
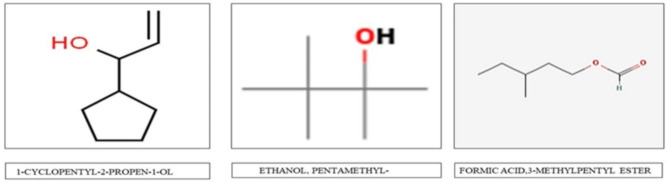
Chemical structure of bioactive compound isolated from acetone extract of *Casearia tomentosa* fruit detected by GC-MS analysis

Phytochemical analysis of the acetone extract of *C. tomentosa* fruit revealed the present of coumarin, alkaloid and terpenoid. Alcohol, Alkane, Aldehyde, Anhydrides, Aromatics, Carbo-Acids, Esters functional groups are detected in FT-IR analysis (Fig. 1) and groups are represented in Table 12. GC-MS analyses of the active component denoted the present of 3 major compounds in the acetone extract of *C. tomentosa* fruits (Fig. 2) that are 1-cyclopentyl-2-propen-1-ol, formic acid,3-methyl-pentyl ester and ethanol, pentamethyl- (Fig. 3).

**Table 12:** Functional groups found in FT-IR analysis of acetone extractive of *Casearia tomentosa* fruit

Sl. No	Peak value (cm^− 1^)	Bonds	Classification	Groups
1	3321.78	OH	Alcohol	HO-R-OH
2	2852.20	CH	Alkane	Cyclohexyl
3	2922.59	CH stretching in Fermi resonance	Aldehyde	Ph-CHO
4	1740.44	C = O	Anhydrides	C-COOCO-C
5	1455.03	Ring	Aromatics	P-disubstituted ring
6	1228.43	C-O	Carbo-Acids	C-CX-COOH
7	1050.05	C-O-C	Esters	COO

**Fig. 4:**
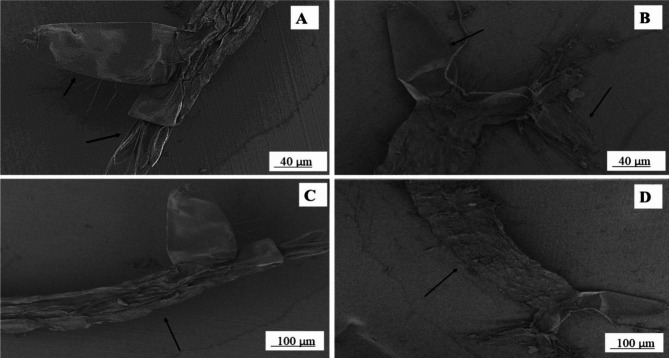
Comparative pictorial presentation before and after treatment of acetone extract on *Culex quinquefasciatus* A represents respiratory siphon of untreated *Culex quinquefasciatus.* B represents respiratory siphon of treated *Culex quinquefasciatus.* C represents abdomen of untreated *Culex quinquefasciatus.* D represents abdomen of treated *Culex quinquefasciatus* (arrow represents the affected area)

**Fig. 5:**
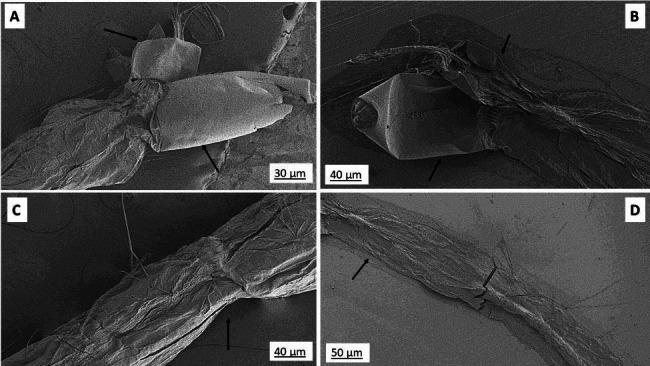
Comparative pictorial representation before and after treatment of acetone extract on *Aedes albopictus* larva. A represents respiratory siphon of untreated *Aedes albopictus.* B represents respiratory siphon of untreated *Aedes albopictus.* C represents abdomen of untreated *Aedes albopictus.* D represents abdomen of treated *Aedes albopictus* (arrow represents the affected area)

FE-SEM studies revealed that the larval abdomen degraded on treatment with the aforementioned extract. Moreover, the anal gills and respiratory siphon showed considerable distortion from their normal state when compared to the control in case of *Ae. albopictus.* With respect to *Cx. quinquefasciatus* the total larval body was damaged as well as the caudal region showed marked changes. (Figures 4 and 5)

## Discussion

Today, synthetic insecticides are most frequently utilised to get rid of larvae in water bodies and adults in the air due to their quick action and accessibility. The use of synthetic chemicals to reduce mosquito populations, however, has recently failed due to the development of insecticide resistance [37]. Hence, pesticide resistance and environmental friendly development have led to an increase in the usage of plant extracts. Plant extracts have a wide range of biological effects on pests, including ovicidal, larvicidal, pupicidal, insect growth regulator, insect enzyme inhibitor effects [38]. This may be as a result of different phytochemicals found in plants that may be interacting together to elicit such responses. Botanical pesticides are biocompatible and rarely develop resistance against the pests because of the synergistic activity of complex biomolecules thereby reducing long-term environmental impacts as seen in synthetic pesticide use [39].

In the current investigation, the acetone extract of *C. tomentosa* fruits demonstrated effective larvicidal ability against both *Cx. quinquefasciatus* and *Ae. albopictus*. Many studies on acetone extracts of various plant particles exhibiting larvicidal properties have been documented [40]. Acetone extract of *Operculina turpethum* leaves showed 100% mortality at 160 ppm against first instar larvae of *Anopheles stephensi* after 72 h of exposure [41]. The acetone extract of *Holoptelea integrifolia* leaves demonstrated larvicidal action, with the maximum mortality being seen at 400 ppm after 72 h against 3rd Instar larvae against *Cx. vishnui* group [42]. Acetone extract of *Acyranthes aspera* foliages exhibited 95% motility at 100 ppm after 72 h of exposure in case of the 3rd instar larvae of *Cx. vishnui* group [43]. However, in this present experiment, 100 ppm was found to cause 100% larval mortality at 24 h of exposure in case of *Cx. quinquefasciatus* and at 72 h of exposure in *Ae. albopictus* larvae. This result is showing significantly higher larval mortality than some other investigations with the acetone extract of different plant with different parts. *Acorus calamus* leaf extract of hexane showed lowest values of LC_50_ 151.86 ppm and 174.70 ppm against *Ae. aegypti* and *Cx. quinquefasciatus* respectively [44]. This investigation showed the lowest LC_50_ value of 35.05 against *Cx. quinquefasciatus* in 2nd instar larvae and 37.69 against 1st instar larvae of *Ae. albopictus* after 72 h of treatment.

*Cx. quinquefasciatus*, however, was found to be more affected than *Ae. aegypti* in a trial with two kinds of *Cyperus esculentus* (black and yellow) [45]. This study also proved that *Cx. quinquefasciatus* was more sensitive to the acetone extract of fruits of *C. tomentosa* than *Ae. albopictus*.

Pharmacological investigations demonstrated that this genus have therapeutic uses in treating inflammation, wounds, infections from fungus and bacteria, tonsilitis, ulcers, fissures, abdominal pain etc. [15]. But there had been no previous record of insecticidal property of this genus, therefore this study claims a novel source of mosquitocide with respect to *Cx. quinquefasciatus* and *Ae. albopictus*.

It is important to note that in semi-field studies, acetone extract showed impressive larvicidal activity against *Cx. quinquefasciatus and Ae. albopictus*, with the highest larvicidal efficacies observed on the third day of treatment. The active ingredient was showing 100% percent mortality rate at a very lose dose as compared to solvent extractive. The acetone extracts of fruits of *C. tomentosa* revealed strong larvicidal effects on *Cx. quinquefasciatus* and *Ae. albopictus*, and showed a decreased level of toxicity towards non-target organisms. In the pupa, the acetone extract also produced encouraging outcomes. Since pupae do not feed, their mortality may indicate the presence of a contact toxin. Here the acetone extract behaves like a contact toxin that kills larvae and pupae. The cuticle of an arthropod acts as its first line of defence against contact insecticides [46]. To enter the neurological system, acetone extract from *C. tomentosa* fruit must first pass through the exoskeleton or cuticle of mosquito. Although it has been established that cuticle alterations, such as thickening, can limit or even stop the penetration of contact insecticides-a phenomena that was originally identified in the 1960s [46–49]. In mature *Anopheles funestus*, the development of a resistance mechanism against pyrethroid is mostly caused by cuticle thickening. The mean cuticle thickness of the tolerant and intolerant specimens was 2.13 μm (SD ± 0.10 μm) and 2.33 μm (SD ± 0.22 μm), respectively, with a mean difference of 0.20 μm [50]. This can potentially increase the efficacy of metabolic detoxification since thicker cuticles slow down the pace at which pesticides are absorbed. The cuticles of larvae of *Chironomus* sp. measure about 3 μm in thickness which is more than cuticular thickness of mosquito species [51]. In the context of pesticide resistance, few studies have directly described modifications in the cuticle of the main arboviral vectors of the genus *Aedes* [52, 53]. The survival of *Chironomus circumdatus* and *Toxorhynchites splendens* seems to be attributed to changes in cuticular thickness between target and non-target organisms. The body weight of 3rd instar larva of *Toxorhynchites splendens* (30 mg) is significantly higher (P < 0.05) than that of *Culex quinquefasciatus* and *Aedes albopictus* (1.6 mg on average). Effective dose of an insecticide always depends on the body weight of target organism. However, the extract was only effective against that particular target that is *Cx. quinquefasciatus* and *Ae. albopictus*. *Toxorhynchites splendens* and *Chironomus circumdatus*, two non-target organisms, are not harmed by contact with the chemicals in the acetone extract. The ineffectiveness of acetone extract in the two non-target species could be attributed to cuticle thickness and body weight. *Acorus calamus* leaf extract of hexane showed clear morphological changes in the siphon, anal papilla, and abdomen region under bright microscope in *Ae. aegypti* and *Cx. quinquefasciatus* [44]. In this investigation clear morphological degradations such as enlarged and flattened abdominal region in breadth, damaged body parts and flattened thoracic region have been observed. In the thoracic and abdominal region of the treated larvae there is no existence of clear segmentation. This demonstrates that acetone extract is responsible for the deformities and mortality of mosquito larva. However, the precise mechanism by which the acetone extract kills the mosquito larva is unclear and requires further extensive study.

In the present study, the HO-R-OH group of alcohol, C-CX-COOH group of carbo acids, Ph-CHO group of aldehyde, Cyclohexyl group of alkane, P-disubstituted ring group of aromatics, and the COO group of esters were all observed using FT-IR spectroscopy. The phytosteriod compounds from leaf extract of *Solanum nigrum* showed the presence of esters group in FT-IR spectroscopy and these compounds have larvicidal property against *Culex vishnui* group and *Anopheles subpictus*. [54] Three pure compounds 1-cyclopentyl-2-propen-1-ol, formic acid,3-methyl-pentyl ester and ethanol, pentamethyl- are isolated in GC-MS analysis from acetone extractive of the fruits of *C. tomentosa* showing significant larvicidal efficacy. The aforementioned phytocompounds from *C. tomentosa* fruits have demonstrated efficacy against *Cx. quinquefasciatus* and *Ae. albopictus*, making them a promising candidate for use as a novel natural larvicidal and pupicidal agent. In the past, isolated active components from the fruit pericarp of *Alangium salvifolium* extracted in ethyl acetate [1] and *Cuscuta chinensis* seeds extracted in chloroform: methanol [4] was reported to have larvicidal effects against *Cx. quinquefasciatus*. Before commercial production and application, a thorough field test and assessment of the physiological mechanism of toxicity should be conducted.

## Conclusion

The current study emphasized on the potential larvicidal and pupicidal properties of the acetone extractive of a novel source of *C. tomentosa* fruits against *Cx. quinquefasciatus* and *Ae. albopictu*s and revealed their chemical constituents, 1-cyclopentyl-2-propen-1-ol, formic acid,3-methyl-pentyl ester and ethanol, pentamethyl- as the most promising compounds with very high selectivity against *Cx. quinquefasciatus* and *Ae. albopictus* and nearly no toxicity to non-targets like *Chironomus circumdatus* and *Toxorhynchites splendens* larvae. FESEM study also visibly discovered morphological alteration in the treated sample from the control one. But, the exact mechanism by which the extract is causing death of mosquito larva is not clearly recognized and further investigation is required. Therefore, we can conclude that the isolated compounds are a better alternative for the field control of *Cx. quinquefasciatus* and *Ae. albopictus* larvae with ecologically safe larvicidal and pupicidal property.

## Data Availability

All experimental data are true to our knowledge. For FT-IR and GC-MS analyses the above said databases has been used which can be provided at any instances.
